# RNPS1 stabilizes NAT10 protein to facilitate translation in cancer via tRNA ac^4^C modification

**DOI:** 10.1038/s41368-023-00276-7

**Published:** 2024-01-22

**Authors:** Xiaochen Wang, Rongsong Ling, Yurong Peng, Weiqiong Qiu, Demeng Chen

**Affiliations:** 1 Center For Translational Medicine, the First Affiliated Hospital, Sun Yat-sen University, Guangzhou, China; 2 Institute for Advanced Study, Shenzhen University, Shenzhen, China

**Keywords:** Microbiology techniques, Head and neck cancer

## Abstract

Existing studies have underscored the pivotal role of N-acetyltransferase 10 (NAT10) in various cancers. However, the outcomes of protein-protein interactions between NAT10 and its protein partners in head and neck squamous cell carcinoma (HNSCC) remain unexplored. In this study, we identified a significant upregulation of RNA-binding protein with serine-rich domain 1 (RNPS1) in HNSCC, where RNPS1 inhibits the ubiquitination degradation of NAT10 by E3 ubiquitin ligase, zinc finger SWIM domain-containing protein 6 (ZSWIM6), through direct protein interaction, thereby promoting high NAT10 expression in HNSCC. This upregulated NAT10 stability mediates the enhancement of specific tRNA ac^4^C modifications, subsequently boosting the translation process of genes involved in pathways such as IL-6 signaling, IL-8 signaling, and PTEN signaling that play roles in regulating HNSCC malignant progression, ultimately influencing the survival and prognosis of HNSCC patients. Additionally, we pioneered the development of TRMC-seq, leading to the discovery of novel tRNA-ac^4^C modification sites, thereby providing a potent sequencing tool for tRNA-ac^4^C research. Our findings expand the repertoire of tRNA ac^4^C modifications and identify a role of tRNA ac^4^C in the regulation of mRNA translation in HNSCC.

## Introduction

Managing head and neck squamous cell carcinoma (HNSCC) is complex, with the primary approach involving surgical procedures and chemoradiotherapy. A significant portion of HNSCC patients succumb to the disease, particularly those with recurrent or metastatic conditions.^1^ Although there is histological evidence of a progression from cellular atypia to various dysplastic stages culminating in invasive HNSCC, the majority of patients receive a late-stage diagnosis without a clinically apparent premalignant lesion.^2^ The limited efficacy of current treatments for patients with HNSCC is evident in the elevated mortality and morbidity rates.^3^ As a result, there is a need for more efficacious therapies targeting HNSCC. Notably, we and others have revealed that targeting dysregulated RNA modification enzymes, such as PCIF1, METTL1 and METTL3, responsible for mRNA m^6^Am, mRNA m^6^A and tRNA m^7^G modification, offers promising strategies for HNSCC treatment.^4–7^ This encourages us to delve deeper into the role of RNA modification in driving HNSCC tumorigenesis and progression.

N4-acetylcytidine (ac^4^C) is commonly recognized as a conservative chemical modification found on both tRNA and rRNA. Moreover, ac^4^C is associated with the development, progression, and prognosis of various human diseases, including cancer.^8,9^ The key enzyme responsible for ac^4^C as an epitranscriptomics modification is N-acetyltransferase 10 (NAT10).^10^ However, the mechanisms underlying the interaction between protein partners and NAT10, as well as the binding proteins that regulate NAT10, remain unexplored. Therefore, in this study, we conducted co-immunoprecipitation (co-IP) combined with liquid chromatography tandem mass spectrometry (LC-MS/MS), leading to the discovery of a key interacting protein, RNA-binding protein with serine-rich domain 1 (RNPS1), that significantly affects the stability of NAT10 protein. Previous research on RNPS1 has primarily focused on its role as a general activator in pre-mRNA splicing.^11–13^ However, its involvement in stabilizing other proteins remains unexplored. Furthermore, our understanding of precise role of RNPS1 in tumorigenesis, is still limited. In this study, we investigated the potential of RNPS1 as a novel target for HNSCC.

Furthermore, current high-throughput detection methods for ac^4^C modification are predominantly based on the antibody-based acRIP-seq,^14–16^ and the limitations of this approach restrict researchers to identifying modification sites within broad regions, without pinpointing specific ac^4^C positions on RNA. Currently, antibody-free methods such as fluorine-assisted metabolic sequencing (FAM-seq) exist.^17^ However, the pro-metabolite used in this method, sodium fluoroacetate, is highly toxic and has lethal effects on most mammals and birds,^18^ rendering it challenging to fully exploit this method in in vivo experiments. Moreover, although FAM-seq no longer relies on antibodies, the positional information of detected ac^4^C still manifests as enrichment peaks, lacking precision down to individual nucleotide positions. While chemical-based sequencing methods like ac^4^C-seq with single-nucleotide resolution have been developed,^10^ they primarily target the most abundant rRNA within total RNA, falling short in their ability to detect tRNA, a relatively scarce RNA rich in rare bases and with shorter single-stranded lengths (76~93 nt).^19^ Therefore, in this study, we pioneered the development of tRNA reduction and misincorporation sequencing (TRMC-seq), a single-nucleotide resolution sequencing method tailored for tRNA, providing a robust tool for advancing research in this field.

In conclusion, our research has elucidated the molecular mechanisms through which RNPS1 functions as a pro-tumorigenic factor in HNSCC. It underscores the crucial role of tRNA-ac^4^C in cancer initiation and progression, and introduces a robust sequencing method for the study of tRNA-ac^4^C, known as TRMC-seq. Furthermore, our findings present a novel therapeutic target for HNSCC prognosis and treatment.

## Results

### RNPS1 interacts with NAT10 in HNSCC

**Fig. 1:**
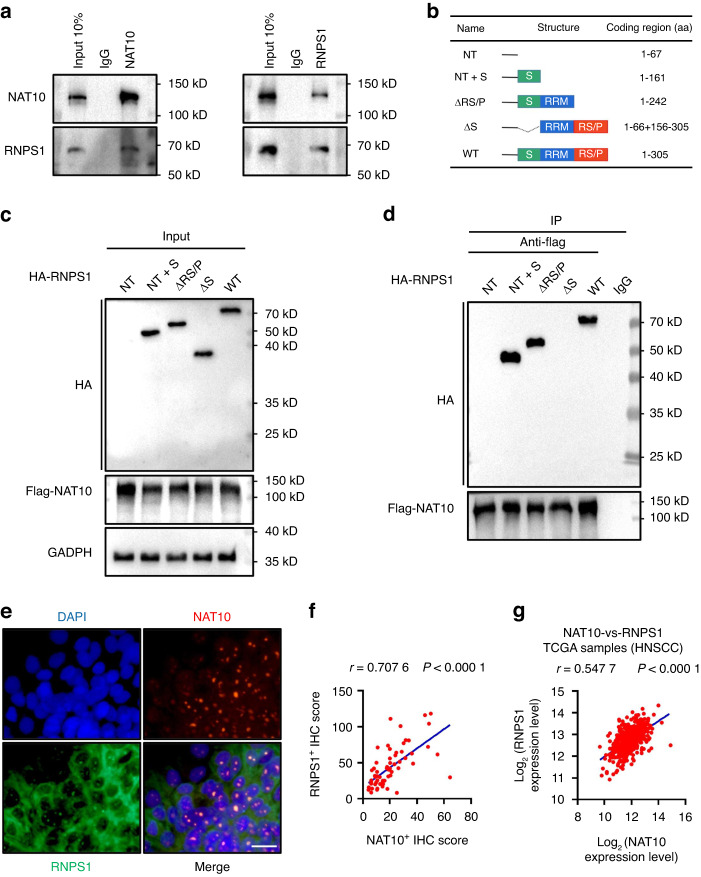
RNPS1 interacts with NAT10 in HNSCC. **a** Association of endogenous NAT10 with RNPS1 in SCC-15 by co-immunoprecipitation (co-IP) with anti-NAT10 antibody or anti-RNPS1 antibody. Anti-IgG antibody was used as a negative control. The experiment was independently replicated twice. **b** Strategy of RNPS1 variant proteins for mapping interaction domains with NAT10. **c** The expression of RNPS1 variant proteins after pcDNA3.1-HA-RNPS1 variant proteins and pICE-FLAG-NAT10-siR-WT were co-transfected into SCC-4 cells. **d** SCC-4 cells were co-transfected with NAT10-Flag and HA-RNPS1 variant proteins as indicated. The NAT10-RNPS1 complex was immunoprecipitated with anti-Flag antibody and detected with anti-HA antibody. Anti-IgG antibody was used as a negative control. **e** Immunofluorescence colocalization staining of NAT10 (red) and RNPS1 (green) expression in SCC-15 cells. The nuclear is counterstained with DAPI (blue). **f** The protein expression of NAT10 and RNPS1 was correlated in HNSCC patient tissues from the Hospital of Stomatology, Sun Yat-sen University. *r* = 0.707 6, *P* < 0.000 1 by Spearman correlation analysis. **g** The correlation plot of NAT10 and RNPS1 expression level in HNSCC using TCGA dataset. *n* = 502, *r* = 0.547 7, *P* < 0.000 1 by Spearman correlation analysis

Current research has identified the significant role played by NAT10 in various cancers.^20,21^ To discover potential NAT10 associated proteins, we performed co-immunoprecipitation (co-IP) to pulldown endogenous NAT10 in SCC-15 cells. The product was run in a gel for a short distance then cut out and digested for a selective and sensitive high-performance liquid chromatography tandem mass spectrometry (LC-MS/MS) analysis. NAT10 was identified in the eluates by LC-MS/MS from SCC-15 cells pulldown by anti-NAT10 antibody but not IgG control, indicating the successful pulldown of NAT10 from cell extracts. To identify the unique NAT10 associated proteins in HNSCC cells, proteins that appeared in the IgG and NAT10 KD control groups were then subtracted. In total, LC-MS/MS of the co-IP proteins identified 18 different proteins (Supplementary Table 1). In particular, we found RNA-binding protein with serine-rich domain 1 (RNPS1) were enriched among these 18 candidates (Supplementary Table 1). To test that, we pulled them down from SCC-15 cell extracts using anti-NAT10 or anti-RNPS1 antibodies. Indeed, the pulldown in each IP experiment contained the other protein (Fig. 1a). We also perform Coomassie brilliant blue to confirmed the presence of RNPS1 band in the gel (Supplementary Fig. 1a, b). To map the domains of RNPS1 that are responsible for its interaction with NAT10. We constructed multiple plasmids to express different domains of RNPS1 with HA (hemagglutinin) tag, including full-length RNPS1 (WT), RNPS1 N-terminal region (NT), a serine-rich domain (S domain) in the N-terminal region (NT+S), RNPS1 without an arginine/serine/proline-rich domain (RS/P domain) in the C-terminal region (ΔRS/P) and RNPS1 without S domain (ΔS) in SCC-4 cells (Fig. 1b). We found RNPS1-NT+S and RNPS1-ΔRS/P, interacted with NAT10 (Fig. 1c, d), suggesting that S domain of RNPS1 was responsible for the interactions with NAT10. Furthermore, double immunofluorescence staining for NAT10 and RNPS1 clearly demonstrated that NAT10 and RNPS1 co-localized in the nucleoli (Fig. 1e) of SCC-15 cells. While NAT10 is predominantly located in the nucleoli, we noticed a broader expression pattern of RNPS1 (Fig. 1e). In addition, a significant positive correlation between NAT10 and RNPS1 protein expression was identified by IHC staining in HNSCC patients (Fig. 1f). Using TCGA database, we found mRNA expression of NAT10 and RNPS1 positively correlated in HNSCC (Fig. 1g). Overall, our experimental results substantiate that RNPS1 is an interacting protein of NAT10 in HNSCC.

### RNPS1 fulfills a pivotal function in the progression and metastasis of HNSCC

**Fig. 2:**
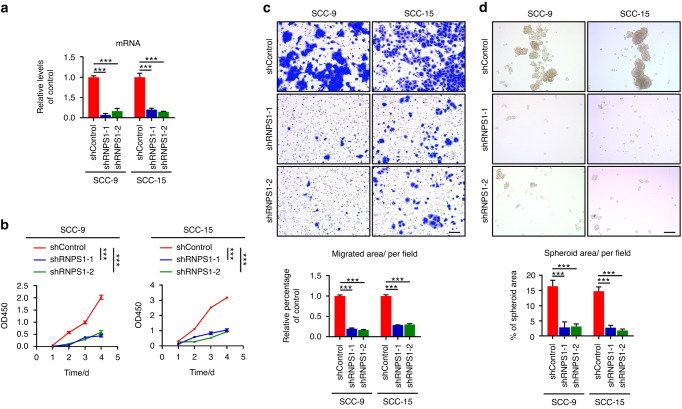
RNPS1 fulfills a pivotal function in the progression and metastasis of HNSCC. **a** qPCR showed that RNPS1 was knocked down by shRNA in SCC-9 and SCC-15. The experiment was independently replicated three times. Data are represented as mean ± standard deviation (SD). ***P < 0.001 by One-way ANOVA. **b** Cell proliferation assay for SCC-9 and SCC-15 cells between the shControl, shRNPS1-1 and shRNPS1-2. Data are represented as mean ± SD. **P < 0.01 and ***P < 0.001 by Two-way ANOVA. **c**, **d** Migration (**c**) and sphere formation (**d**) between the shControl, shRNPS1-1 and shRNPS1-2 in HNSCC cell lines. In the migration assay (**c**), Mitomycin C (MMC) was added to inhibit the effect of cell proliferation. These experiments were independently replicated three times. Data are represented as mean ± SD. ***P < 0.001 in (**c**) and (**d**) by One-way ANOVA

**Fig. 3:**
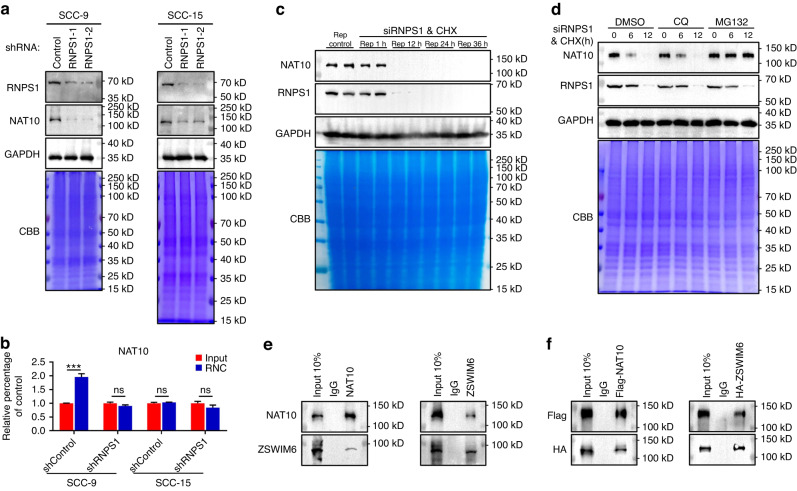
RNPS1 ensures the stability of NAT10 by inhibiting the ubiquitination of NAT10. **a** The expression of NAT10, RNPS1 and GAPDH in control group and KD groups of HNSCC cell lines. GAPDH and CBB staining were used as loading control. **b** The content of NAT10 mRNA at the ribosome combined stages and input stages after KD of RNPS1. Data are represented as mean ± SD. ***P < 0.001 by Two-way ANOVA. **c** WB image and analysis showing NAT10 protein expression level in SCC-15 cells for serial RNPS1 knockdown and cycloheximide (CHX, 100 μg/mL) treatment durations. WB of GAPDH and Coomassie brilliant blue staining were used as loading control. **d** WB showing expression level in cells treated with 100 μg/mL cycloheximide (CHX) for the indicated durations in the presence of RNPS1 knockdown with 20 μmol/L chloroquine (CQ) or 10 μmol/L MG132. WB of GAPDH and Coomassie brilliant blue staining were used as loading control. **e** Association of endogenous NAT10 with ZSWIM6 in SCC-15 by co-IP with anti-NAT10 antibody or anti-ZSWIM6 antibody after RNPS1 knockdown and MG132 treatment. Anti-IgG antibody was used as a negative control. **f** Co-IP assay of Flag-NAT10 and HA-ZSWIM6 in 293T cells, which were transiently co-transfected with Flag-NAT10 and HA-ZSWIM6 plasmids and treated with RNPS1 knockdown and MG132

Given the interaction between RNPS1 and NAT10 and the existing research indicating NAT10’s impact on tumor occurrence and progression in various cancers,^20,21^ we aimed to investigate whether RNPS1 affects the advancement and metastasis of HNSCC. We initially generated stable RNPS1 knockdown (KD) cell lines for SCC-9 and SCC-15 using two distinct lentiviral constructs (Figs. 2a and 3a). Functionally, depletion of RNPS1 resulted in the suppression of cell proliferation and migratory capacity (Fig. 2b, c). To assess whether RNPS1 is critical for the stem cell-like properties of HNSCC cells, we conducted sphere-forming assays. Our findings demonstrated that depletion of RNPS1 significantly reduced the tumor sphere formation frequency of SCC-9 and SCC-15 cells (Fig. 2d). Additionally, we detected an increased number of apoptotic cells in RNPS1 KD cells compared to control cells (Supplementary Fig. 2a). To corroborate these findings, we employed The Cancer Genome Atlas Head-Neck Squamous Cell Carcinoma (TCGA-HNSC) cohort for an assessment of RNPS1 expression and its prognostic significance. Indeed, RNPS1 expression exhibited a substantial upregulation in HNSCC samples and was linked to cancer progression (Supplementary Fig. 2b–e). In conclusion, our research results suggest that RNPS1 is pivotal for the tumorigenic characteristics of HNSCC.

### RNPS1 ensures the stability of NAT10 by inhibiting the ubiquitination of NAT10

We noticed that RNPS1 KD led to decreased level of NAT10 (Fig. 3a). We then examined our RNC-seq data and found the mRNA level and translation ratio of NAT10 was not affected after RNPS1 KD (Fig. 3b), indicating destabilization of NAT10 protein after RNPS1 KD. To explore how NAT10 is degraded after RNPS1 KD, we first treated cells with RNPS1 siRNA. We then determined the half-life of NAT10 protein (Fig. 3c). Subsequently, we used either the lysosomal inhibitor chloroquine (CQ) or proteasome inhibitor MG132 to dissect the pathway that is responsible for NAT10 degradation. Our data demonstrated that NAT10 degradation is mediated by proteasome (Fig. 3d and Supplementary Fig. 3a). Since the ubiquitin–proteasome system (UPS) is responsible for degrading most proteins, we wondered which E3 ubiquitin ligase was recruited to NAT10 protein. At this point, we looked into our LC-MS/MS data to search for the E3 ubiquitin ligase candidates. We found ZSWIM6, an E3 ubiquitin ligase,^22,23^ was associated with NAT10 (Supplementary Table 1). We confirmed this interaction by co-IP experiments between NAT10 and ZSWIM6 both endogenously and exogenously (Fig. 3e, f and Supplementary Fig. 3b, c) after RNPS1 KD. Furthermore, our data showed RNPS1 KD significantly increase level of ubiquitination of NAT10 (Supplementary Fig. 3d, e), which can be blocked by knockdown of ZSWIM6 (Supplementary Fig. 3f). In summary, our data suggested that RNPS1 can stabilize NAT10 by protecting its associated with E3 ubiquitin ligase ZSWIM6.

### NAT10 interacts with RNPS1 to regulate tRNA ac^4^C modifications

**Fig. 4:**
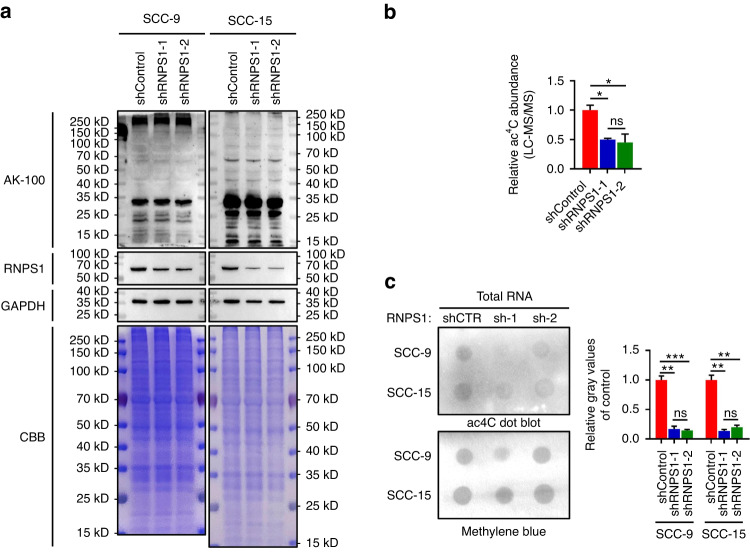
Knockdown of RNPS1 results in reduced levels of ac^4^C in the RNA of HNSCC. **a** The lysine acetylation levels of protein detected by WB with anti-acetylated-Lysine (Ac-K2-100) antibody in RNPS1 KD cells. GAPDH and CBB staining were used as loading control. **b** The bar chart of relative ac^4^C abundance of RNA by LC-MS/MS from SCC-15 cell line. The samples are total RNA from Control and RNPS1 KD groups. *n* = 2 independent biological replicates. Data are represented as mean ± SD. *P < 0.05 by One-way ANOVA. **c** Dot blot (left) and relative gray values (right) showed the ac^4^C level of total RNA in Control and RNPS1 KD groups with methylene blue staining as loading control. *n* = 2 independent biological replicates. Data are represented as mean ± SD. **P < 0.01 and ***P < 0.001 by One-way ANOVA

**Fig. 5:**
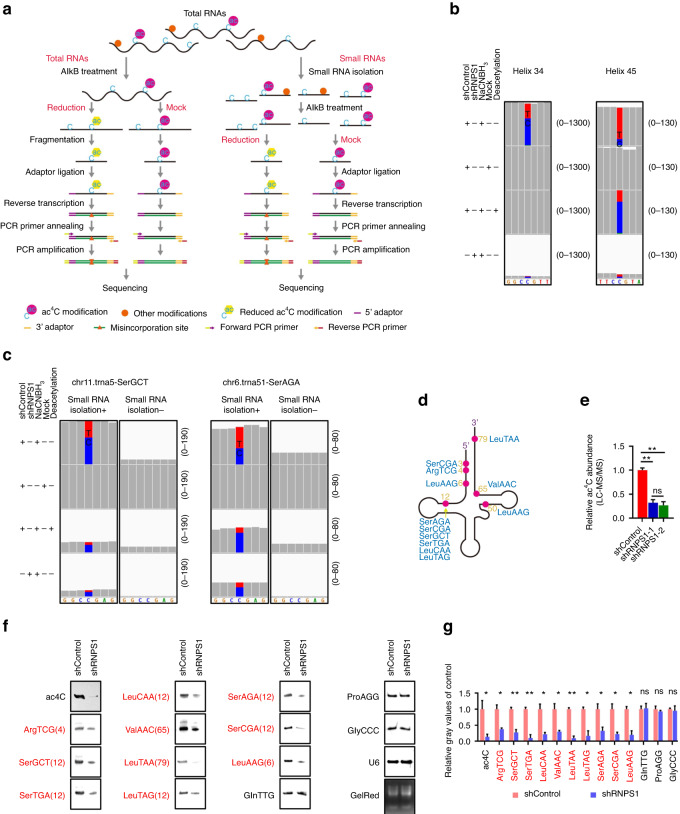
NAT10 interacts with RNPS1 to regulate tRNA ac^4^C modifications. **a** Schematic diagram of total RNA reduction and misincorporation sequencing (left) and tRNA reduction and misincorporation sequencing (TRMC-seq) (right). **b** Misincorporation rates in total RNA from SCC-15 cells are shown for known sites in 18S rRNA. blue letters C and bars, cytidine; red letters T and bars, thymidine; green letters A, adenosine; orange letters G, guanosine. The number on the right indicates the number of nucleotides in the ordinate. The specimens were detected by total RNA reduction and misincorporation sequencing. **c** Representative misincorporation sites of tRNASer(GCT) and tRNASer(AGA) in small RNA and total RNA from SCC-15 cells were detected by TRMC-seq or total RNA reduction and misincorporation sequencing. Small RNA isolation+, detected by TRMC-seq; Small RNA isolation-, detected by total RNA reduction and misincorporation sequencing. The number on the right indicates the number of nucleotides in the ordinate. **d** A total of 7 different ac^4^C sites were found in 10 tRNA isoacceptors by TRMC-seq. **e** The bar chart showed relative ac^4^C abundance of small RNA by LC-MS/MS between Control and RNPS1 KD groups of SCC-15 cells. *n* = 2 independent biological replicates. Data are represented as mean ± SD. **P < 0.01 by One-way ANOVA. **f**, **g** NWB & NB (**f**) and relative gray values (**g**) showed ac^4^C modification levels of RNA and expression of non-ac^4^C tRNAs and ac^4^C tRNAs (red font) between Control and RNPS1 KD groups. NB of U6 snRNA and GelRed staining were used as loading control. The numbers in brackets represent the ac^4^C sites. *n* = 2 independent biological replicates. Data are represented as mean ± SD. *P < 0.05 and **P < 0.01 in (**g**) by unpaired Student’s *t* test

NAT10 has known to participate in acetylation of critical oncogene proteins.^24,25^ Hence, we conducted acetylated-lysine (Ac-K-100) assay to investigate whether NAT10, a downstream protein regulated by RNPS1, is involved in protein acetylation in HNSCC cells. However, our Ac-K-100 results showed no discernible difference between control and KD of RNPS1 HNSCC cells (Fig. 4a). Subsequently, we shifted our focus towards RNA acetylation modifications. A remarkable discovery was that following RNPS1 depletion, both SCC-9 and SCC-15 cells exhibited a significant reduction in ac^4^C modifications in total RNA (Fig. 4b, c). We then modified chemical-based ac^4^C-seq method to examine RNA ac^4^C in control, mock, deacetylation, and shRNPS1 HNSCC groups^10^ (Fig. 5a). Our sequencing data identified ac^4^C modification in 18S rRNA helix 34 and helix 45, two well-known sites that have been reported previously^10^ (Fig. 5b), supporting the legitimacy of our approach. However, sequence analysis results indicate the absence of qualified sites on mRNA, and the existence of ac^4^C modification in eukaryotic cell mRNA remains a subject of contention.^10,26^ Since NAT10 is well-known for its catalyzing the formation of ac^4^C on tRNA^Ser^ and tRNA^Leu^,^10,27,28^ we then analyzed ac^4^C on tRNA in HNSCC cells. Surprisingly, we found 13 ac^4^C sites in 9 tRNA isoacceptors, including ac^4^C_12_ on tRNA^Ser(GCT)^; ac^4^C_79_ on tRNA^Leu(TAA)^; ac^4^C_12_ on tRNA^Leu(TAG)^; ac^4^C_12_ on tRNA^Ser(TGA)^; ac^4^C_12_ on tRNA^Ser(AGA)^; ac^4^C_12_ on tRNA^Ser(CGA)^; ac^4^C_4_ on tRNA^Arg(TCG)^; ac^4^C_6_ on tRNA^Leu(AAG)^ and ac^4^C_12_ on tRNA^Leu(CAA)^ in our ac^4^C-seq data (Fig. 5c, d and Supplementary Table 2), suggesting a broader effect of NAT10 on tRNA ac^4^C. As expected, the depletion of NAT10 caused by RNPS1 knockdown resulted in a decreased of misincorporation proportion in the same tRNA ac^4^C sites (Fig. 5c). Bolstered by these finding, we then isolated small RNAs (<200 nt) and performed mass spectrometry and ac^4^C dot blot assays. We found levels of ac^4^C on small RNAs were reduced after RNPS1 KD (Fig. 5e and Supplementary Fig. 4a). Moreover, mass spectrometry results indicated that the decline in ac^4^C of tRNA was more pronounced compared to total RNA (Figs. 4b and 5e). In order to find the specific sites of acetylation modification on tRNA, we developed a method more suitable for tRNA reduction and misincorporation sequencing (TRMC-seq) (Fig. 5a). Based on this approach, we found ac^4^C on 10 tRNA isoacceptors, including 16 tRNA isodecoders (17 sites) using TRMC-Seq (Fig. 5c, d and Supplementary Table 2). Besides the sites described above, TRMC-seq results revealed several novel sites, including ac^4^C_3_ on tRNA^Ser(CGA)^; ac^4^C_50_ on tRNA^Leu(AAG)^; ac^4^C_65_ on tRNA^Val(AAC)^ (Fig. 5d and Supplementary Table 2), supporting higher sensitivity of TRMC-seq in detecting ac^4^C on tRNAs. To probe the function of ac^4^C modification in tRNA regulation, we compared the expression levels of non-ac^4^C-modified tRNA and ac^4^C-modified tRNA. We found decrease of ac^4^C-modified tRNA levels after KD of RNPS1 compared to non-ac^4^C-modified tRNA (Supplementary Fig. 4b, c). Northern blot results confirmed that KD of RNPS1 led to suppression of ac^4^C-modified tRNA detected by TRMC-seq, including tRNA^Arg(TCG)^, tRNA^Ser(TGA)^, tRNA^Leu(CAA)^ and tRNA^Val(AAC)^ (Fig. 5f, g and Supplementary Fig. 4d). Thus far, our findings have demonstrated that ubiquitination of NAT10, triggered by RNPS1 depletion, mediates the ac^4^C modification levels on tRNA in HNSCC.

### Integrated analysis of multiple ‘omics’ reveal RNPS1 regulation of translation

**Fig. 6:**
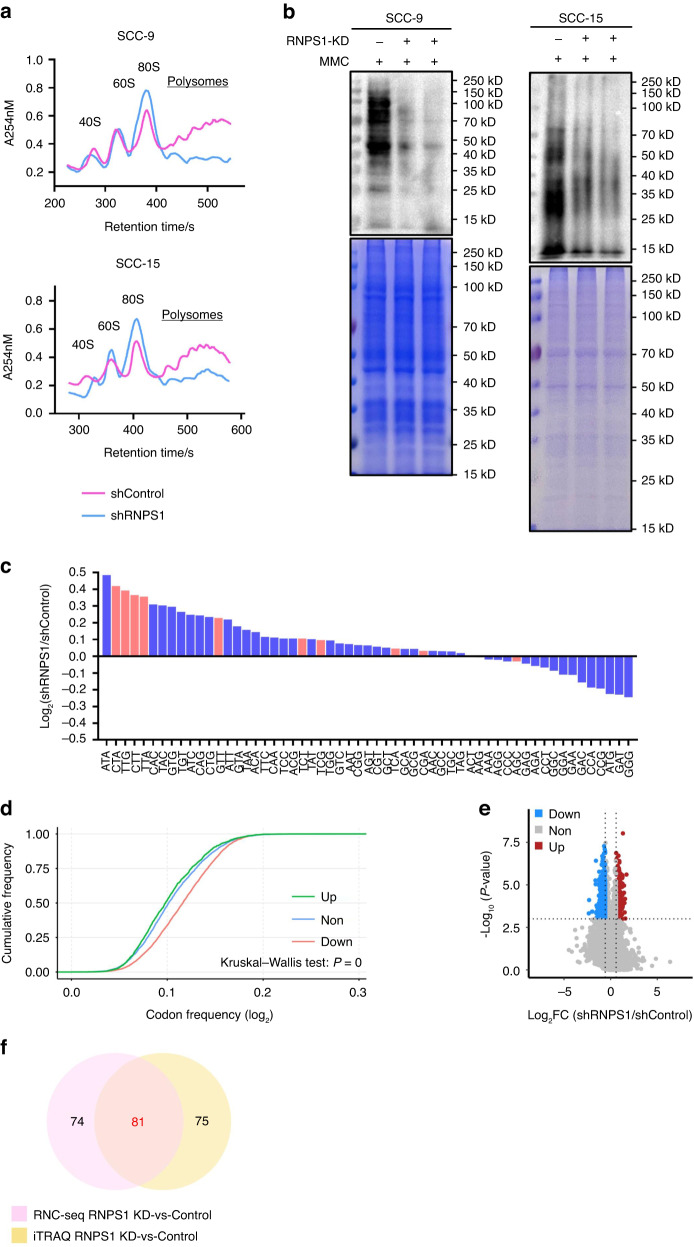
Integrated analysis of multiple ‘omics’ reveal RNPS1 regulation of translation. **a** Polysome profiling of the Control and RNPS1 KD groups in SCC-9 (up) and SCC-15 (down) cell lines. MMC was added to inhibit the effect of cell proliferation. **b** Puromycin assay revealed reduced protein production in RNPS1 KD cells. MMC was added to inhibit the effect of cell proliferation and CBB staining was used as loading control. **c** Codon occupancy bias of RNPS1 KD groups in SCC-15. The ac^4^C tRNA corresponding to the codons were depicted by the red bars. **d** Codon frequency in the CDS region of the genes with increased TE (up, green), decreased TE (down, red) and other genes (non, blue) of RNPS1 KD cells (F). *P* = 0 by Kruskal–Wallis test. **e** Volcano plots of translation ratio (TR) in RNPS1 KD cells. Red dots represent the genes with upregulated TE. Blue dots represent the genes with downregulated TE. *P* values by unpaired Student’s test. **f** Venn diagram of IPA pathway analysis for RNC-seq and iTRAQ in RNPS1 KD cells. pink, RNC-seq (RNPS1 KD-vs-Control); yellow, iTRAQ (RNPS1 KD-vs-Control)

To explore the global translation dynamics by RNPS1, we performed polysome profiling assay to isolate the polysome-associated translated mRNAs from untranslated ones based on sucrose-gradient separation. Both SCC-9 and SCC-15 cells after RNPS1 KD showed clear reduction in polysome peaks compared to the control cells (Fig. 6a). To test whether decreased of polysome peaks reflected a less active translating mRNA, we measured protein synthesis using SUnSET, a puromycin/antibody-based method. Our results revealed inhibition of translation at the protein level after RNPS1 KD in SCC-9 and SCC-15 cells (Fig. 6b). In addition, results from ribosome profiling assays showed that KD of RNPS1 increased the codon-dependent ribosome pause of ac^4^C tRNA, supporting that ac^4^C tRNA modifications mediated by RNPS1-regulated NAT10 was critical for efficient codon recognition during the ribosomal transition of ac^4^C tRNA decoding codons (Fig. 6c). In addition, codon frequency analysis demonstrated that the mRNAs with increased translation efficiency (TE) possess significantly lower frequencies of ac^4^C-modified tRNA decoding codons (Fig. 6d).

To fully understand how multifaceted aspects of translation are controlled by RNPS1 in HNSCC, we determined the translatomic and proteomic profiles of the RNPS1 KD and control SCC-15 cells. We first used ribosome nascent-chain complex-bound mRNA sequencing (RNC-seq) to compared the translating mRNA profile between control and RNPS1 KD samples. We found 1878 differentially expressed translational active mRNA after RNPS1 KD, including 1481 downregulated translating mRNA and 397 upregulated translating mRNA (Fig. 6e and Supplementary Table 3). Canonical pathway analysis on the downregulated translating genes using ingenuity pathway analysis (IPA) revealed significant enrichment in pathways involved in molecular mechanisms of cancer, IL-6 signaling, IL-8 signaling, Rac signaling, PTEN signaling, actin cytoskeleton signaling and HGF signaling (Supplementary Table 4). To determine the proteomic profiles of HNSCC cells after RNPS1 KD, we conducted isobaric tags for relative and absolute quantitation (iTRAQ) assay. In 6 190 total proteins identified in our iTRAQ datasets, we detected 621 down-expressed proteins after RNPS1 KD (Supplementary Table 5). IPA analysis demonstrated that the ablation of RNPS1 in HNSCC cells down-expressed the proteins involved in cholesterol biosynthesis, acute phase response signaling and IL-8 signaling (Supplementary Table 6). Importantly, following RNPS1 ablation, among the 155 pathways enriched for downregulated genes in RNC-seq, 81 pathways (52.26%) overlapped with those enriched in the iTRAQ (Fig. 6f, Supplementary Tables 4 and 6). This suggests that RNPS1 influences the translation process in HNSCC through NAT10.

### Reduced expression of RNPS1 attenuated malignancy in vivo

**Fig. 7:**
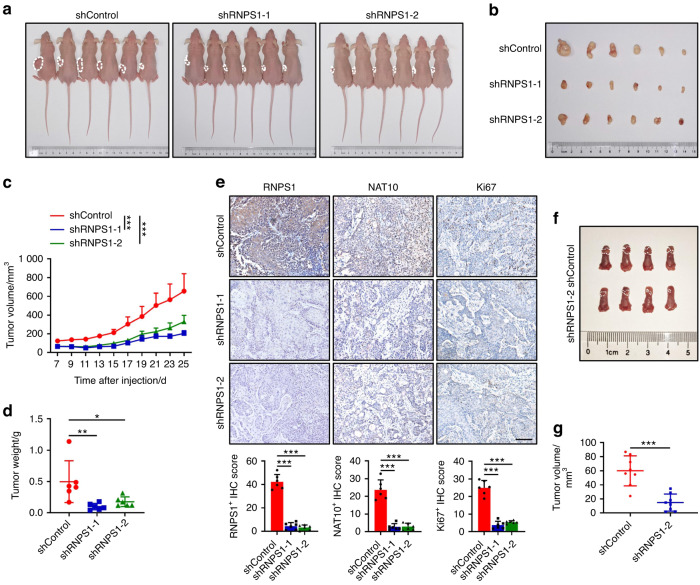
Reduced expression of RNPS1 attenuated malignancy in vivo. **a** Pictures of tumor-bearing nude mice injected with SCC-15 cells of shControl, shRNPS1-1 and shRNPS1-2 groups. *n* = 6 mice in each group. **b** Pictures of tumor formation of xenograft in nude mice. *n* = 6 mice in each group. **c** The tumor growth curve of RNPS1 knockdown cells was compared with vector control cells. *n* = 6 mice in each group. Data are represented as mean ± SD. ***P < 0.001 by Two-way ANOVA. **d** Weights of tumors in three groups were measured using electronic scales. *n* = 6 mice in each group. Data are represented as mean ± SD. *P < 0.05 and **P < 0.01 by One-way ANOVA. **e** Representative IHC staining and IHC Score of RNPS1 (left), NAT10 (middle) and Ki67 (right) in HNSCC between different treatment groups. Scale bars are 100 μm. Data are represented as mean ± SD. ***P < 0.001 by One-way ANOVA. **f** Representative image of xenografted SCC-15 cell line including shControl and shRNPS1-2 in tongue of nude mice. The white dotted line indicates tumor boundary. *n* = 8 mice in each group. **g** Tumor volume (mm^3^) of orthotopic transplantation tumor between shControl and shRNPS1-2. *n* = 8 mice in each group. Data are represented as mean ± SD. ***P < 0.001 by unpaired Student’s *t* test

We finally sought to validate whether RNPS1 could suppress the malignant phenotype of HNSCC in vivo. Nude mice were subcutaneously injected with control and two RNPS1 KD SCC-15 cells, and after 4 weeks, the mice were euthanized for sample collection. Our experimental results demonstrate that, compared to the control group, the deficiency of RNPS1 in SCC-15 cells significantly inhibited tumor growth (Fig. 7a, b), leading to a marked reduction in tumor volume and weight (Fig. 7c, d). Additionally, through Ki67 immunohistochemical (IHC) staining, we observed a pronounced suppression of HNSCC’s proliferative capacity following RNPS1 depletion (Fig. 7e), which aligns with our prior in vitro findings. Furthermore, we conducted orthotopic implantation experiments in nude mice to assess the tumorigenic and metastatic potential of RNPS1-stable KD SCC-15 cells in an in vivo setting. The tumor volume in the KD group was notably smaller than that in the control group (Fig. 7f, g and Supplementary Fig. 5a). The depletion of RNPS1 likewise leads to a diminished capability for cellular proliferation and metastasis to the cervical lymph nodes (Supplementary Fig. 5b).

## Discussion

HNSCC represents a lethal malignancy with limited therapeutic options for late-stage patients,^29–31^ underscoring the pressing need to identify novel therapeutic targets for HNSCC treatment. RNA modifications have been reported to enhance tumor cell proliferation, invasion, metastasis, and immune evasion in various malignancies, including HNSCC.^4,32–34^ Consequently, we conducted research centered around the NAT10 protein, known for catalyzing the formation of ac^4^C in RNA. Our investigations led to the discovery of RNPS1, a protein that binds to NAT10 and regulates its stability, preventing its degradation through ubiquitination by the E3 ubiquitin ligase ZSWIM6. In this study, we observed that depletion of RNPS1 results in the ubiquitination and degradation of NAT10 protein, leading to a reduction in ac^4^C modification on tRNAs in HNSCC, which subsequently initiates tRNA degradation and significantly impedes the protein translation process within tumor cells. Rapid proliferation of tumor cells relies on the swift synthesis of proteins, making an active translation process indispensable.^35^ Effective inhibition of the translation process in HNSCC can impact its proliferation. Additionally, analysis of TCGA database suggests a high expression of RNPS1 in tumor tissues. Therefore, targeting RNPS1 as a novel therapeutic approach may offer new strategies for HNSCC patients.

Typically, due to the elevated incidence of antibody-binding false positives and the inability of binding fragments to precisely resolve to individual nucleotides, antibody-based sequencing methods utilizing next-generation sequencing are perceived as less dependable for the detection of RNA modifications. Nevertheless, chemical-based sequencing methodologies adeptly tackle both of these challenges.^10,36,37^ Currently, although chemical-based high-throughput sequencing methods capable of detecting ac^4^C sites on RNA have been proposed,^10^ there is still a dearth of efficient and precise targeted sequencing methodologies tailored to highly structured, extensively modified, and relatively low-abundance RNA molecules such as tRNA. In addressing this issue, we isolated tRNAs, augmented their relative abundance, and employed the purified bacterial demethylase AlkB along with an optimized AlkB mutant to proficiently eliminate interference from tRNA methylation and other modifications. This approach (TRMC-seq) enabled a more efficient and precise tRNA sequencing process. By doing so, we are better equipped to identify ac^4^C-tRNA in HNSCC and discover several previously unreported tRNA-ac^4^C sites. Due to differences in mRNA sequences among various genes, there is variation in the codon composition. Consequently, tRNAs carrying anticodons that match these codons exhibit specificity, resulting in specific tRNA selection in the translation process between different genes.^38–40^ Indeed, an analysis of codon utilization for the most downregulated genes in our data reveals a higher percentage of codons corresponding to tRNAs bearing ac^4^C modifications. Our IPA analysis of these downregulated genes revealed their enrichment in pathways such as IL-6 signaling, PTEN signaling and IL-8 signaling, which have been documented to impact HNSCC tumor growth and metastasis.^41–43^ Building upon the preceding context, we have ascertained that in HNSCC, RNPS1 depletion exerts its tumor growth and metastasis inhibitory function by selectively suppressing the translation of genes involved in signaling pathways like IL-6 signaling.

## Materials and methods

### Experimental model and subject details

#### Cell culture and generation of mutant cell lines

SCC-4, SCC-9, SCC-15 and 293T were purchased from the American Type Culture Collection (ATCC). SCC-4, SCC-9 and SCC-15 were maintained in 1:1 mixture of Dulbecco’s modified Eagle’s medium and Ham’s F12 medium (DMEM/F12, Gibco, C11330500BT) at a 37 °C incubator containing 5% CO_2_. All cell line media were supplemented with 10% fetal bovine serum (FBS, Gibco, 10270-106) and 1% penicillin/streptomycin (Gibco, 15140-122).

For stable KD of NAT10 and RNPS1 in HNSCC cells, we used lentivirus to integrate shRNA into the host cell lines. Briefly, the shRNAs targeting NAT10 or RNPS1 were cloned into pLKO.1 plasmid, and then co-transfected into 293T cells with packaging vector psPAX2 and enveloped vector pMD2.G using Lipofectamine 2000 reagent (Invitrogen, 11668019). Subsequently, the medium supernatant containing lentivirus was collected and added into the medium of SCC-9 or SCC-15 cells with 10 μg/mL Polybrene (YEASEN, 40804ES76). After 48 h, the positive clones were screened with 2.5 μg/mL puromycin (Beyotime, ST551-250 mg). For mapping of RNPS1 domains that interact with NAT10, we constructed various truncated RNPS1 with HA tag into pcDNA3.1 vector and co-transfected with pICE-FLAG-NAT10-siR-WT into SCC-4 through Lipofectamine 2000 reagent. 24–48 h later, cell lysate was immunoprecipitated with anti-Flag antibody (Proteintech, 80010-1-RR) and Protein A/G magnetic beads (Thermo Fisher Scientific, 88803). The NAT10-RNPS1 complex was detected with anti-HA antibody after immunoblotting. For ubiquitination assay, RNPS1 KD and/or ZSWIM6-KD cells transfected with the indicated plasmids pICE-FLAG-NAT10-siR-WT and pCDH-MYC-Ubiquitin were cultured with 10 μmol/L MG132 (Selleck, S2619) and lysed. The supernatants were subjected to immunoprecipitation and immunoblot analysis with the indicated antibodies as described below.

#### Human subjects

HNSCC tissues and adjacent tissues were collected from patients with HNSCC who underwent surgery at the Hospital of Stomatology, Sun Yat-sen University. This study received the informed written consent from all patients before the experiment.

#### Animal studies

All animal studies were approved by the Institutional Animal Care and Use Committee, Sun Yat-sen University (IACUC, SYSU). All animals were housed under specific pathogen-free conditions and handled in the Laboratory Animal Center, Sun Yat-sen University. The approval number is SYSU-IACUC-2020-000437 and SYSU-IACUC-2021-000122. BALB/c-nu/nu (Application No: 20191230-00047) mice were purchased from the Laboratory Animal Center, Sun Yat-sen University.

#### Establishment of orthotopic transplanted tumor model in nude mice

For orthotopic transplant assay, nude mice (BALB/c-nu/nu) maintained under specific pathogen-free conditions were used. Five weeks after birth, the nude mice were anesthetized with 87.5 mg/kg Ketamine/12.5 mg/kg Xylazine intraperitoneally, routinely disinfected, and injected with a total 50 μL mixture of cell suspension and phosphate buffer saline (1:1, approximately 5 × 10^5^ cancer cells) into the tongue of the mice. The whole operation was completed within 1 h after the preparation of single cell suspension. After inoculation, the mice were kept in the sterile laminar flow room, and the vitality of the mice were observed for 24 h to determine whether they were affected with anesthetics. For subcutaneous tumorigenesis, differences in the number of cancer cells (1 × 10^6^), the other procedures are similar to those described above.

### Method details

#### RNA extraction and real-time PCR

Cell pellets were collected and then subjected to total RNA extraction using AG RNAex Pro Reagent (Accurate Biotechnology, AG21102) according to the manufacturer’s instructions. Next, in accordance with the instructions, real-time PCR was performed using PerfectStart® Green qPCR SuperMix (TransGen Biotech, AQ601), by a Bio-Rad CXF96 real-time system (Bio-Rad, USA). Applying an internal control, the relative quantity was calculated.

#### Total protein extraction and Western blot analysis

Protein lysates were isolated in lysis buffer and followed by the addition of sample loading buffer (4Abio, 4APA008-15), separated by 10% SDS polyacrylamide gel electrophoresis (EpiZyme, PG11X) and transferred onto PVDF membranes (Merck Millipore, IPVH00010). The membranes incubated with primary antibodies against: NAT10 (Santa Cruz, [sc-271770] or Proteintech, [13365-1-AP], 1:2 000), RNPS1 (Proteintech, 10555-1-AP, 1:2 000), HA (Abcam, ab9110, 1:4 000) and GAPDH (Cell Signaling Technology, 2118S, 1:2 000). Enhanced chemiluminescence (ECL) method with appropriate species-specific horseradish peroxidase-conjugated secondary antibodies (Proteintech, anti-rabbit [SA00001-2] 1:4 000, anti-mouse [SA00001-1] 1:4 000) were used to visualize the blots.

#### Cell proliferation assay

CCK-8 assay (DOJINDO, CK04-500T) was conducted to determine the cell proliferation. According to the manufacturer’s instructions, cell suspension was added into 96-well plate (0.01 × 10^6^–0.02 × 10^6^ cells per well). The absorbance at 490 nm was measured after addition of CCK-8 reagent to cells.

#### Cell migration assay

Cell migration assay was carried out using Cell Culture Insert (Merck millipore, MCHT06H48). Cells were put into the chamber containing 600 mL medium for 4–6 h of culture in cell incubator. And DMEM containing 20% FBS was served as a stimulating factor. Then cells were wiped out carefully and stained with crystal purple (Beyotime, C0121). 1 h later, the chamber was moved to the empty 24-well plate, followed by an observation under the microscope.

#### Dot blot

RNAs including total RNA and small RNA were extracted and enriched (small RNA was enriched through MirVana miRNA Isolation Kit (Invitrogen, AM1561)) before dot blots were performed using rabbit monoclonal anti-ac^4^C antibodies (Abcam, ab252215). Briefly, equal amounts of diluted RNAs were denatured at 90 °C for 5 min and 4 °C for 1 min. After that, RNAs were added into an Amersham Hybond N+ membrane (GE Healthcare, RPN203B) and crosslinked twice with 1 200 mJ for 25–50 s in the Stratalinker 2400 UV Crosslinker (Stratalinker, USA). Membrane was blocked with 5% non-fat milk in 1 × PBST for 1 h at room temperature and then was inducted overnight with anti-ac^4^C antibody in 1% non-fat milk at 4 °C. After several washing steps with 0.1% PBST, horseradish peroxidase coupled secondary antibody was applied at 4 °C overnight. Membrane was developed with Chemiluminescent Imaging System (Tanon 5200 SF, China).

#### Tumor sphere formation assays

To establish tumor spheres, cells were seeded onto ultra-low attachment 6-well plate at 10 000 cells per mL and cultured seven days in the tumor sphere medium as previously described.^44^ The cells were cultured for seven days, during which serum-free media were changed every other day until the spheres formed. Three dishes were used for each group and all experiments were repeated three times.

#### Flow cytometric analysis of apoptosis

Cells were collected, washed, spun down and labeled with fluorescein isothiocyanate (FITC)-Annexin V and propidium iodide using the FITC Annexin V Apoptosis Detection Kit (DOJINDO, AD10). Apoptotic cells were analyzed by using FACS LSRFortessa flow cytometry (BD Biosciences, USA). Graphs show percentage of viable cells, dead cells, early apoptotic cells, and late apoptotic cells.

#### tRNA reduction and misincorporation sequencing (TRMC-seq)

The isolated small RNAs’ other domain modifications were removed by AlkB and AlkB-mut, which quenched with 0.5 mol/L EDTA to a final concentration of 5 mmol/L EDTA. Next the AlkB-treated RNAs were recovered by Oligo Clean & Concentrator kit (ZYMO RESEARCH, D4060) and divided into three groups: NaCNBH_3_ (treated with NaCNBH_3_ and without pre-treatment of mild alkali), Deacetylation (treated with NaCNBH_3_ and pre-treatment of mild alkali), Mock (without any treatment). Then the RNAs were precipitated with 2.5 volume cold ethanol at −20 °C for at least four hours and desalted with 75% cold ethanol. After precipitation, the RNAs were ligated with 3′ adapters, hybridized with reverse transcription primers and ligated with 5′ adapters. Ligated RNAs were reverse transcribed using TGIRT-III (InGex, TGIRT50) and performed PCR amplification. Subsequently, the products were purified by QIAquick PCR Purification kit (QIAGEN, 28104) and circularized by the splint oligo sequence forming the single strand circle DNA, followed by sequencing with MGISEQ-2000 (Fig. 5a). For total RNA reduction and misincorporation sequencing, we fragmented RNA and skipped the step of small RNA isolation. We then carried out the remaining steps as described in the TRMC-seq (Fig. 5a).

#### RNC-seq

The total ribosome bound and unbound mRNAs were separated by gradient centrifugation. The content and quality of RNA were detected by gel electrophoresis and Nano-300 Micro Spectrophotometer. The library preparations were subsequently sequenced on a platform of BGISEQ-500. TR was calculated by dividing the ribosome combined fragments signals by the input RNA-seq signals.

#### Polysome assay

For polysome assay, cells were incubated with PBS containing 100 μg/mL cycloheximide (CHX, Sigma-Aldrich, C7698). 15 min later, cells were harvested with polysome cell extraction buffer. We then separated the supernatant, measured the OD value and added the supernatant on the top of 11 mL 10%–50% sucrose-gradient tube. After centrifugation (36 K r/min for 2–2.5 h at 4 °C with max break), the samples were analyzed and plotted by BR-188 Density Gradient Fractionation System (Brandel, USA).

#### Puromycin assay

For puromycin assay, also known as SUnSET assay,^45^ cells were first incubated with puromycin (1 μmol/L final concentration) for 30 min, then harvested for protein extraction. 15 µg protein were loaded onto a SDS polyacrylamide gel for electropherosis. The following steps are carried out like regular Western blot analysis described above. The concentration of the first antibody-anti-Puromycin is 1:2 000 (Merck millipore, MABE342).

#### Northwestern blot (NWB) and Northern blot (NB)

Briefly, 2 or 3 µg total RNA samples were mixed with 2 × RNA loading buffer and denatured at 95 °C for 5 min and 4 °C for 1 min. After 15% UREA-PAGE electrophoresis, the RNAs were transferred onto a positive charged nylon membrane mentioned above. The RNAs on the membrane were crosslinked with UV and then blotted with digoxigenin-labeled probes against tRNAs or U6 snRNA. For Northwestern blot, the RNA containing membranes were blotted with anti-ac^4^C antibody overnight. Finally, the digoxigenin or anti-ac^4^C antibody signals were detected following the Western blot protocol described above.

#### Liquid chromatography tandem mass spectrometry (LC-MS/MS)

Proteins were first separated using SDS polyacrylamide gels. Gels were then stained with Coomassie blue to visualize proteins bands. We then cut the gel bands with proteins and put them into a clean 1.5 mL centrifuge tube. Gel bands were rinsed with ddH_2_O and decolorized with decolorizing solution (50% acetonitrile (Thermo Fisher Scientific, A998-4L), 25 mmol/L ammonium bicarbonate (Thermo Fisher Scientific, 1066-33-7)) to completely white with acetonitrile and vacuum dried. Next, we added dithiothreitol (DTT, Amresco, 0281-BEJ-100G) to the tube and performed reduction reaction at 56 °C for 1 h. After removing DTT, the reduced protein was alkylated by iodoacetamide (IAM, Sigma-Aldrich, I6125-10G) and incubated at room temperature in dark for 45 min. Then ammonium bicarbonate and acetonitrile were successively used for cleaning and decolorization. The dried gel bands were digested with trypsin at 37 °C overnight. Subsequently, formic acid (Thermo Fisher Scientific, 64-18-6) was added to stop the digestion reaction and detected by Q Exactive Mass Spectrometer (Thermo Scientific).

#### Protein digestion and iTRAQ labeling

The proteins were reduced and alkylated according to the method (LC-MS/MS) mentioned above and we purified samples through acetone precipitation. Then Bradford protein assay was used to determine the protein concentration and 50 μg of protein was diluted with 8 mol/L urea in 100 mmol/L TEAB. Trypsin was used for protein digestion. The samples were digested overnight at 37 °C. After that, peptides were desalted and vacuum dried, according to the manufacturer’s protocol. Then the peptides were labeled according to the instructions provided by iTRAQ Reagents-8plex kit (AB SCIEX, 4390812).

#### Mass spectrometry of ac^4^C RNAs

RNA samples were extracted as previously shown. Add 1 μg of RNA samples into the buffer solution, completely enzymolysis the samples into nucleosides at 37 °C under the action of phosphodiesterase (0.002 U/μL; Sigma-Aldrich, P3243), S1 nuclease (180 U/μL; Takara, 2410A) and alkaline phosphatase (30 U/μL; Takara, 2250A), and re extract the enzymatically hydrolyzed sample with chloroform-method. Put the obtained upper aqueous solution into the injection bottle and analyze it via LC-MS/MS. The chromatographic column mainly adopts Waters ACQUITY UPLC HSS T3 C18 column (1.8 μm, 100 mm × 2.1 mm i.d.). At 40 °C, the column flow rate was set to 0.3 mL/min. For mass spectrum, the temperature of the electrospray ion source was set to 550 °C, and the mass spectrum voltage is set to 5 500 V under positive electrospray ionization mode.

#### Histologic evaluation and immunohistochemical staining

For hematoxylin and eosin (H&E) staining, tissues were fixed in formalin, paraffin-embedded, sectioned (5 μm), deparaffinized and stained with H&E staining kit (Solarbio, G1120-3). For IHC staining, sections were deparaffinized and treated with 3% H_2_O_2_ in water for 10 min. Antigen retrieval procedure was conducted to sections with 10 mmol/L citrate buffer (pH 6.0) for 10 min. Next, tissue sections were incubated with the Biocare blocking reagent for 10 min, followed by an overnight incubation at 4 °C with anti-NAT10 (Santa Cruz, sc-271770 1:200), anti-RNPS1 (Proteintech, 10555-1-AP, 1:200), anti-Ki67 (Novus, NB500-170, 1:200), anti-PCK (pan-Cytokeratin, Santa Cruz, sc-8018, 1:200). Slides were then incubated with goat anti-rabbit horseradish peroxidase-conjugated secondary antibodies for 30 min at room temperature, treated with 3,3′‐diami‐nobenzidine and counterstained with hematoxylin. For analysis, the staining intensity was scored and the percentage of positive stained areas of tumor cells per the whole tumor area were calculated.

#### Immunofluorescence staining

The cell climbing sheets were fixed with 4% paraformaldehyde in PBS overnight and stained with the following primary antibody: anti-NAT10 (Santa Cruz, sc-271770 1:100) & anti-RNPS1 (Proteintech, 10555-1-AP, 1:100) after permeated with 1% Triton™ X-100 (Sigma-Aldrich, X-100). Then, the antigens were visualized by the corresponding secondary antibody conjugated with DyLight 488 and Fluor 594. The nuclei were counterstained with DAPI (Solarbio, C0065) at 1:1 000 for 1 min. Images were captured with an upright fluorescence microscope (ZEISS, Germany).

### Quantification and statistical analysis

#### Quantification of TRMC-seq

SRNAtools^46^ was used to infer tRNA expressions. In brief, after trimming the adapter and filtering low-quality sequences, the clean sequencing reads were mapped to a reference genome and different small RNA libraries using Bowtie^47^ with a maximum of two mismatch. The mapped reads are used to identify and profile tRNAs. DEseq^48^ was then used to infer the statistical significance of differential expression of tRNAs. As the default, a tRNA is considered to be significantly differentially expressed when the *P* value is ≤0.05 and the fold change is at least 1.5-fold.

#### Analysis of Ribo-seq

RiboToolkit (RiboToolkit: an integrated platform for analysis and annotation of ribosome profiling data to decode mRNA translation at codon resolution) was used to analyze the codon occupancy based on Ribo-seq. In the analyzing process, the clean Ribo-seq sequences were first aligned to rRNAs, tRNA and snRNA to exclude the RPFs coming from rRNA, tRNA and snRNA using Bowtie^47^ with a maximum of two mismatches. Cleaned RPF sequences were then mapped to the reference genome using STAR.^49^ The unique genome-mapped RPFs are then mapped against protein coding transcripts using Bowtie. For codon-based analyses, 5′ mapped sites of RPFs (26–32 nt) translated in 0-frame were used to infer the P-sites and the occupancy on each codon was calculated. The codon occupancy was further normalized by the basal occupancy which was calculated as the average occupancy of +1, +2 and +3 position downstream of A-sites. TE was calculated by dividing the ribosome protected fragments (RPF) signals by the input RNA-seq signals.

#### Other statistical analyses

Statistical analyses were performed using GraphPad Prism 8 software. All data were presented as mean ± SD unless otherwise specified. Statistical significance was determined by *P* < 0.05. For statistical comparisons, unpaired Student’s *t* test (and nonparametric test for two groups), one-way ANOVA analyses (three groups or more groups), two-way ANOVA analyses (three groups or more groups for two factors) and Pearson chi-square test (metastatic lymph node for transgenic mice) were performed. Significance was defined *P < 0.05, **P < 0.01, ***P < 0.001.

### Supplementary information

Figure S1

Figure S2

Figure S3

Figure S4

Figure S5

Table S1. related to Figure 1

Table S2. related to Figure 5

Table S3. related to Figure 6

Table S4. related to Figure 6

Table S5. related to Figure 6

Table S6. related to Figure 6

Table S7. related to Methods

## Data Availability

The raw data including RNC-seq, Ribosome-seq, total RNA reduction and misincorporation sequencing and tRNA reduction and misincorporation sequencing data have been deposited at GSA (accession number HRA002348).
