# Supplementary figures and images for: RNPS1 stabilizes NAT10 protein to facilitate translation in cancer via tRNA ac4C modification
Source: Int J Oral Sci. 2024 Jan 22;16:6. doi: 10.1038/s41368-023-00276-7 (PMC10800354; doi:10.1038/s41368-023-00276-7)

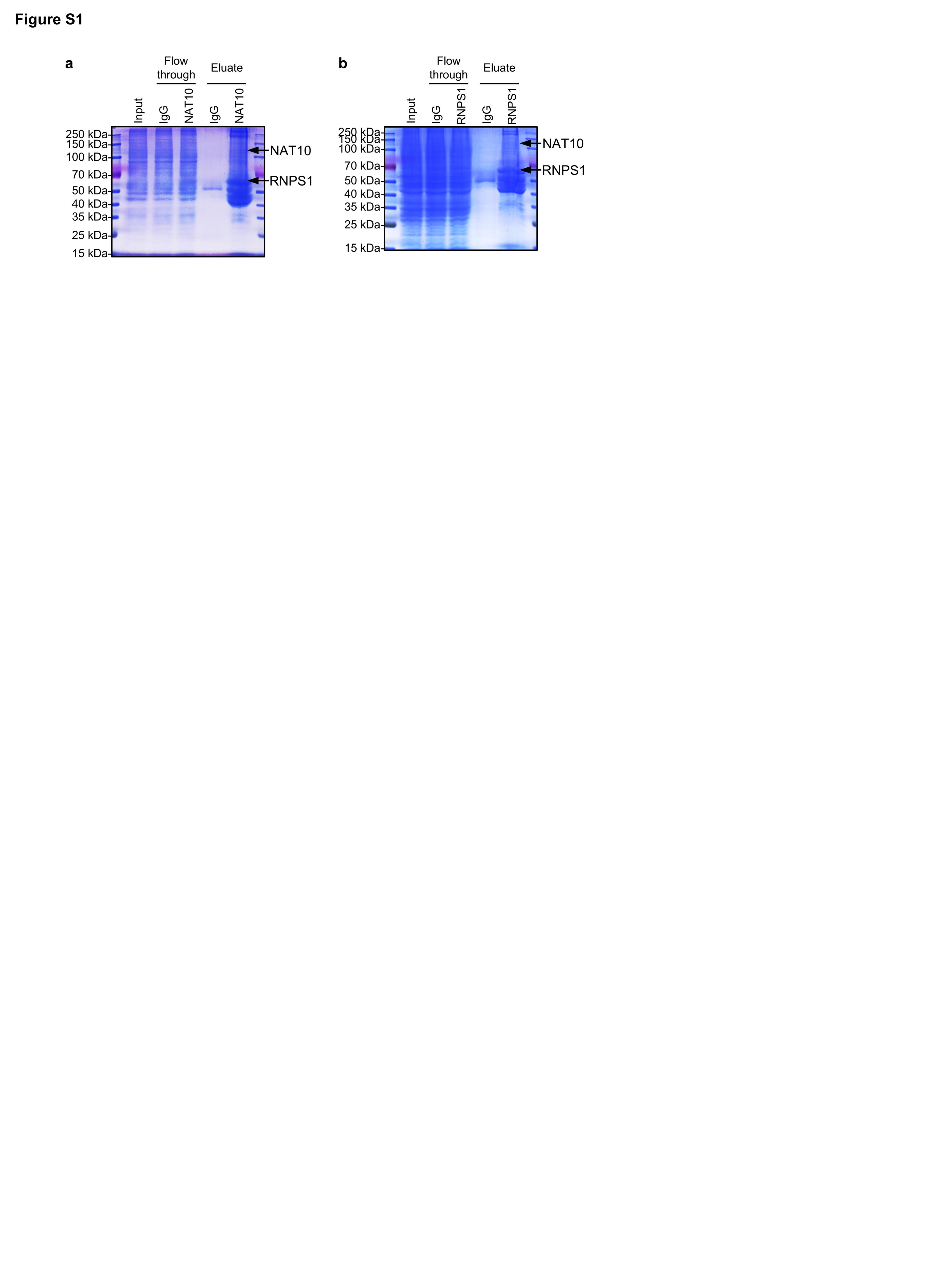

Supplement: Supplementary file 1 — Figure S1 [file 41368_2023_276_MOESM1_ESM.jpg]

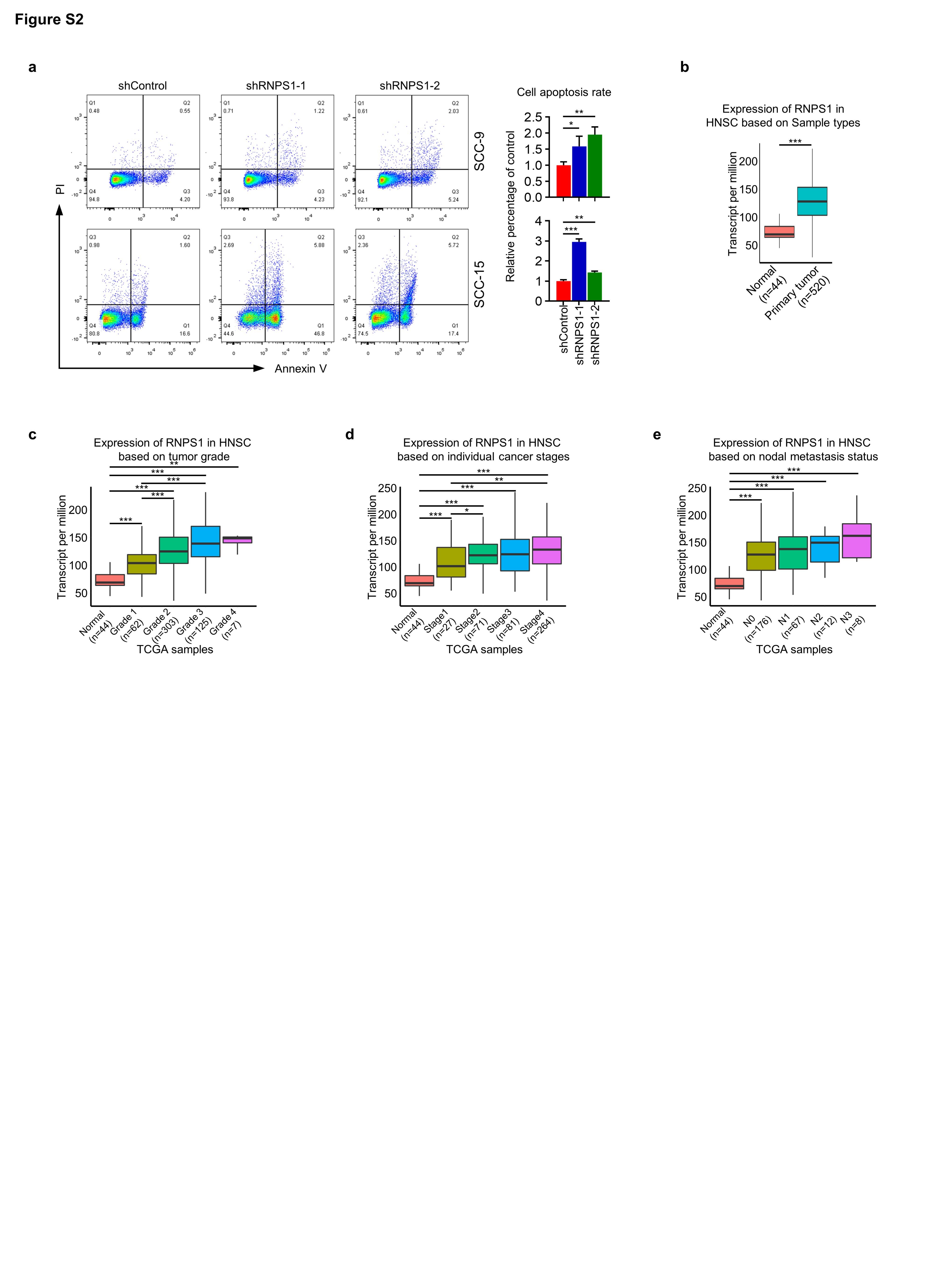

Supplement: Supplementary file 2 — Figure S2 [file 41368_2023_276_MOESM2_ESM.jpg]

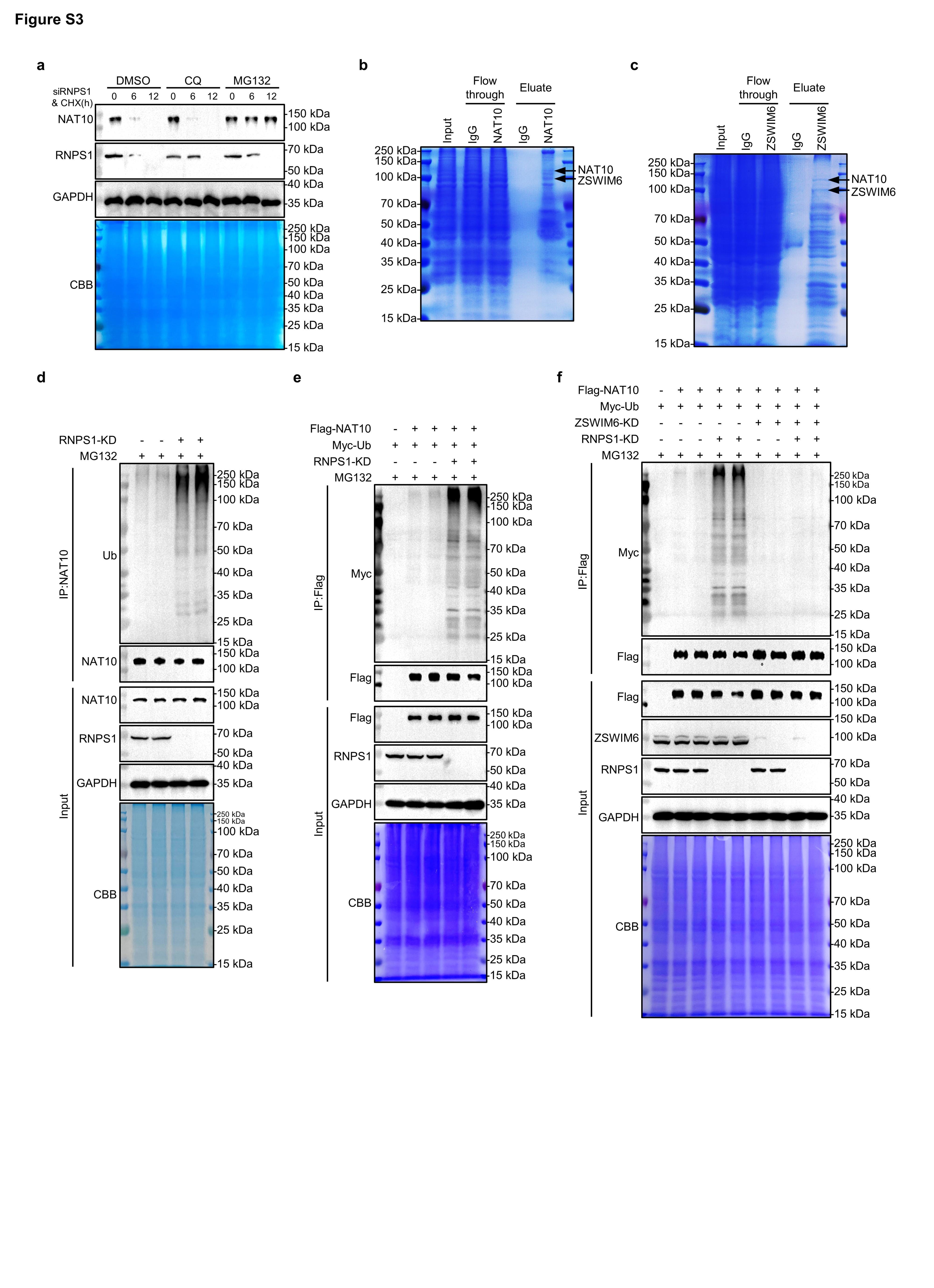

Supplement: Supplementary file 3 — Figure S3 [file 41368_2023_276_MOESM3_ESM.jpg]

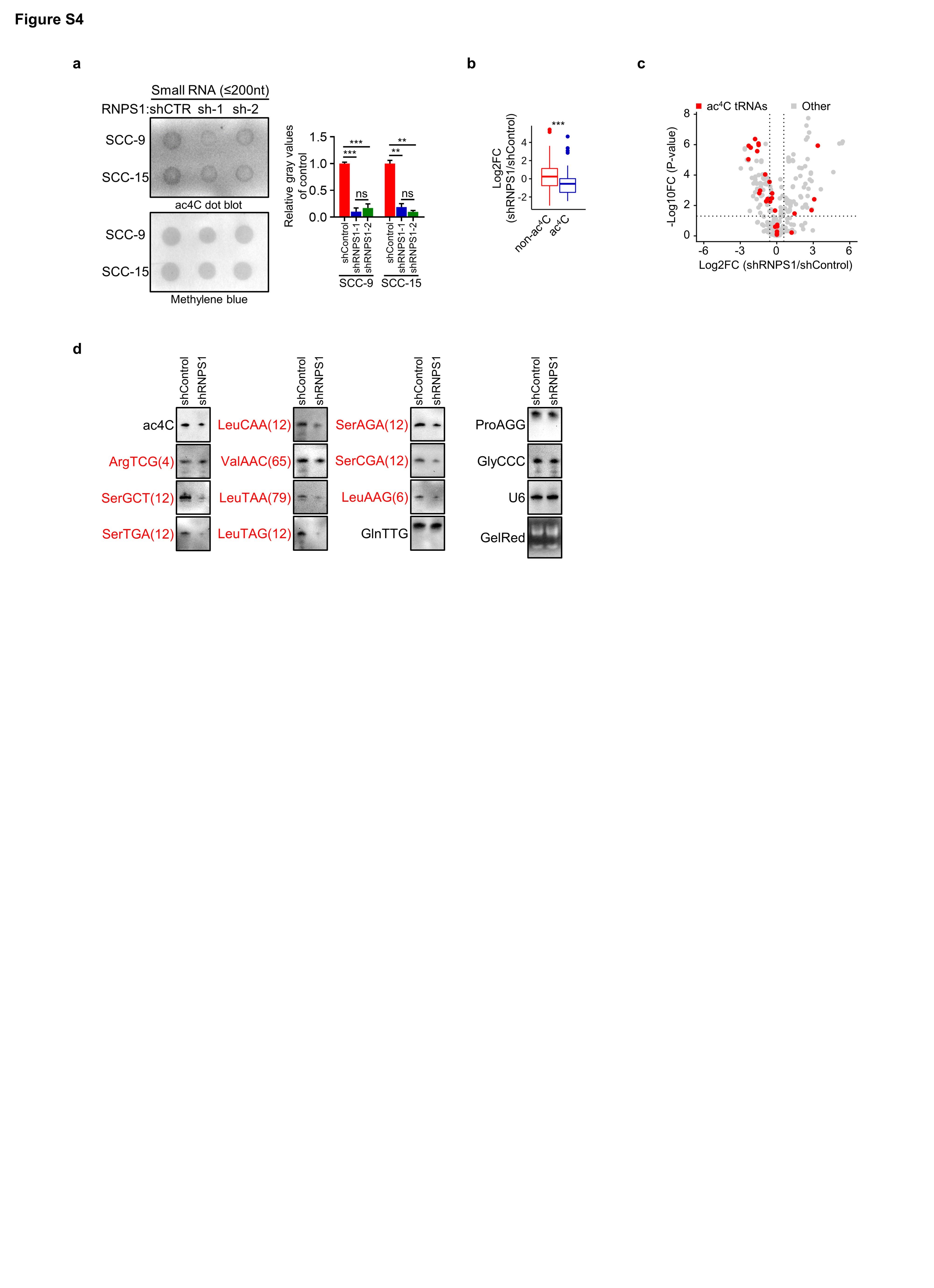

Supplement: Supplementary file 4 — Figure S4 [file 41368_2023_276_MOESM4_ESM.jpg]

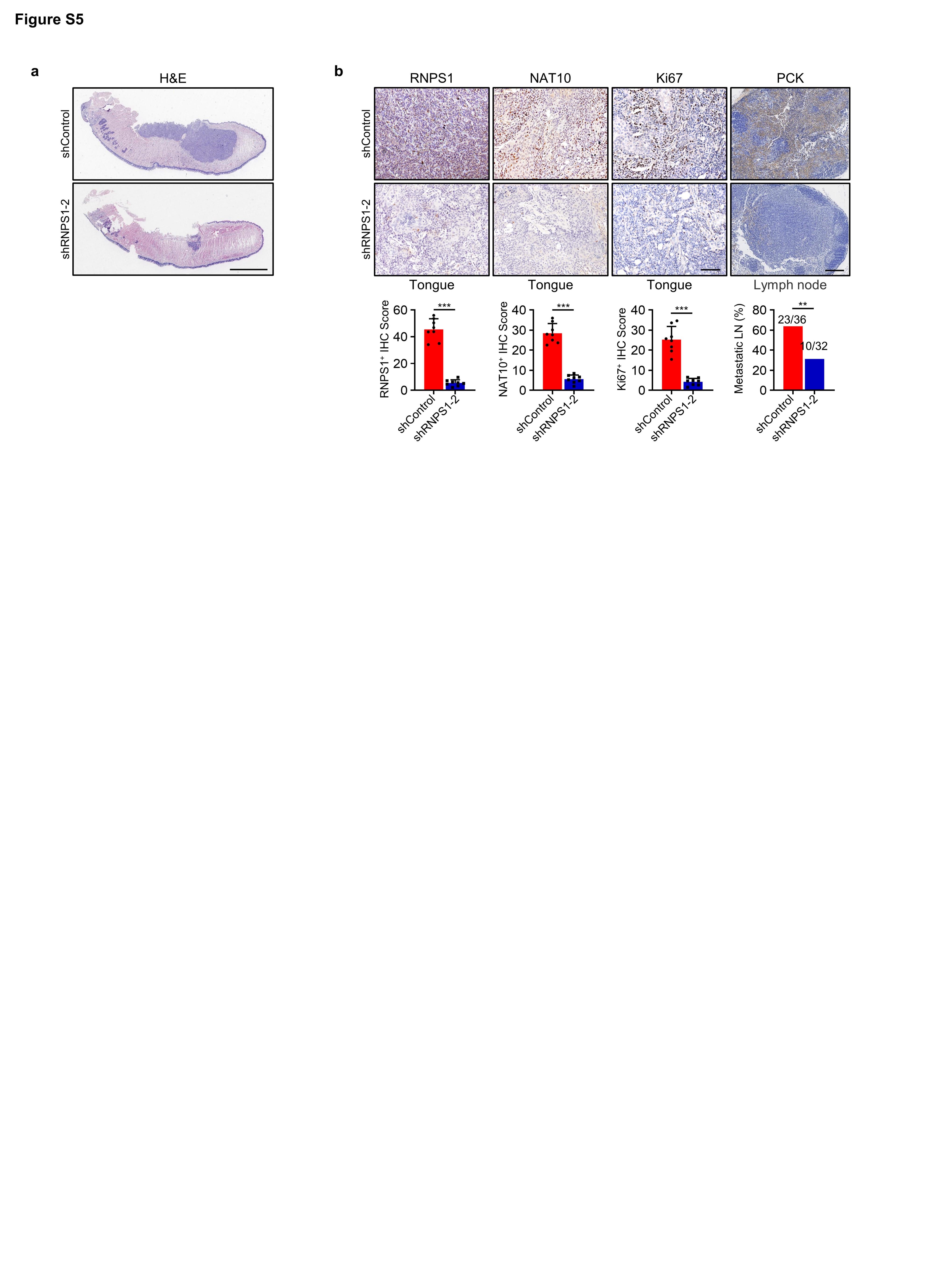

Supplement: Supplementary file 5 — Figure S5 [file 41368_2023_276_MOESM5_ESM.jpg]
